# The role of microbial cell free DNA sequencing in sepsis detection in the neonate

**DOI:** 10.1038/s41390-024-03568-8

**Published:** 2024-09-12

**Authors:** Gergely Toldi, Atif Majid

**Affiliations:** Liggins Institute, University of Auckland, Auckland, New Zealand

## Abstract

A Commentary on “Microbial cell-free DNA-sequencing as an addition to conventional diagnostics in neonatal sepsis”

Bloodstream infections remain a major diagnostic dilemma in neonatal medicine. Due to the non-specific initial presentation of sepsis, prompt commencement of empiric antibiotics is the cornerstone of management and vital for patient survival.^1^ This, however, results in an overuse of antibiotics in the neonatal population, with several known negative implications on the individual patient,^2^ as well as on the population level.^3^ To better balance the identification of patients at risk and our overall use of antibiotics, it is essential to improve our current diagnostic array of bloodstream infections. The current gold standard, blood culture-based methods, remain time-consuming and require a significant blood volume, which is a major issue, particularly in preterm infants. They are also unsuitable to identify obligate anaerobes and intracellular pathogens, including viruses.

There is growing evidence of the diagnostic use of cell-free DNA (cfDNA) from patient samples for various purposes, including feto-maternal and cancer diagnostics, and, more recently, pathogen identification.^4^ cfDNA comprises fragments (approximately 160 bp) of double-stranded deoxyribonucleic acid that are not encapsulated in cells,^5^ and was initially described by Mandel and Metais in 1948, in the plasma of healthy subjects.^6^ Growing evidence suggests that blood samples also harbor circulating microbial cfDNA (mcfDNA). Tong et al reported that more than 95% of mcfDNA originated from bacteria, but eukaryotic and viral sequences were also detected.^7^

While next-generation sequencing (NGS) of mcfDNA in the newborn infant was initially considered as a valuable addition to the existing sepsis diagnostic array, a recent systematic review by Agudelo-Perez et al. concluded that the number of studies was limited in this population, and are at high risk of bias.^8^ The report in this issue of Pediatric Research by Balks et al. highlights some of the limitations of this method in a neonatal cohort.^9^

The authors conducted a single-center, prospective study in neonates receiving a diagnostic workup for suspected late-onset sepsis (LOS), who were admitted during the first 28 days of life to the neonatal intensive care unit between September 2020 and July 2022. Patients were allocated to one of three groups: (1) blood culture-positive sepsis, (2) blood culture-negative sepsis, and (3) suspected sepsis. A fourth validation group included healthy newborns without clinical signs of infection. The DISQVER NGS platform for the identification of mcfDNA was compared with standard blood cultures. Additionally, since many NGS results, including the validation cohort, had no matching blood culture results, a case-by-case assessment was performed, serving as a composite reference standard. This included (1) blood culture results, where available, (2) results of other routine microbiological or virologic examinations, and (3) the patient’s clinical course.

Overall, 15 out of 46 blood cultures were positive in the study. NGS detected mcfDNA from pathogenic, but also from likely non-pathogenic bacteria, fungi, and viruses, even when blood cultures yield negative results. NGS correctly identified pathogenic organisms in nine out of 15 patients with positive blood cultures, including two with intrinsic resistance to their antibiotic regimen. Importantly, out of the 15 positive blood cultures, NGS produced a negative result in four cases. These included two blood cultures growing S. epidermidis, taken from a patient with a 14-day interval, and two other cultures growing E. coli and K. pneumoniae, respectively. All four cultures were considered to be true bloodstream infections, rather than contamination.

Sensitivity and specificity of NGS based on comparison to blood culture results were calculated for the 46 samples where results for both methods were available and were found to be 73.33% and 74.19%, respectively, with an overall accuracy of 73.91%. Positive percent agreement between NGS results and the composite reference standard was calculated to be 82.61%, while negative percent agreement was 91.43%, with an overall accuracy of 87.93%.

Although it was hypothesized that NGS may improve detection rates in patients who had already been started on antibiotics at the time of sampling, no such benefit of NGS was reported compared to blood cultures. The short half-life of cfDNA or the absence of a focus of infection with continuous release of mcfDNA might explain why an increased detection rate was absent using NGS.

The authors also discuss some further challenges associated with the interpretation of positive NGS results. These include understanding whether the identified pathogen is of acute clinical relevance, or the result of contamination of laboratory processing or reagents, or simply reflects remnants of pathogens already cleared by immune cells and, therefore, of no clinical significance. To ensure appropriate clinical utilization of NGS results, the authors recommend consultation with infectious disease specialists and integration with clinical and microbiological data. Another important limitation of NGS is its inability to provide information regarding antimicrobial susceptibility, although this may improve in the future by expanding the library of common resistance genes that sequencing may identify.

While NGS has the potential to augment the precision of conventional diagnostic techniques, improving the detection of pathogens and targeted treatment approaches in neonatal sepsis, this prospective study did not reveal the major benefits of NGS in the neonatal and preterm population of patients assessed for bloodstream infection. Furthermore, no indication or analysis of associated costs and cost-benefit ratio were provided. There is also no discussion of the turnaround time of samples in comparison with blood cultures, which could heavily depend on local logistic arrangements and infrastructure.

At present, this method could only be recommended in specific, pre-defined clinical scenarios and groups of patients rather than for general use. It does not overcome the major hurdles of blood culture-based diagnostics: prior antibiotic exposure reduces the chances of a positive find, and a significant blood volume (minimum 0.5 mL) is necessary. On the other hand, it might offer advantages over blood cultures in the spectrum of pathogens detected, with the addition of obligate anaerobes and intracellular pathogens.

In summary, while the findings of Balks et al. are promising, further validation of NGS of mcfDNA in neonatal sepsis diagnostics is necessary, and its place and utility in the diagnostic workflow needs to be fine-tuned. Replication and comparison of the current findings with adequately powered, multicenter studies will facilitate this process. In the meantime, interpretation of NGS results requires correlation with clinical data and existing routine diagnostic methods for a comprehensive understanding of the patient’s condition.
